# Case Report: Bexagliflozin as an adjunct decongestive strategy in a cat with congestive heart failure and advanced chronic kidney disease

**DOI:** 10.3389/fvets.2026.1791139

**Published:** 2026-03-13

**Authors:** Byung-Jun Kim, Kun-Ho Song

**Affiliations:** 1 College of Veterinary Medicine, Chungnam National University, Daejeon, Republic of Korea; 2 Hannam Cardiology and Internal Medicine Animal Hospital, Seoul, Republic of Korea

**Keywords:** bexagliflozin, cat, chronic kidney disease, congestive heart failure, SGLT2i

## Abstract

Sodium–glucose cotransporter 2 inhibitors (SGLT2i) have established cardiorenal benefits in human medicine; however, clinical reports describing their use as a decongestive strategy in cats with congestive heart failure (CHF) complicated by advanced chronic kidney disease (CKD) remain limited. This case describes an 11-year-old neutered male British Shorthair cat with advanced CKD (IRIS stage 3–4) that developed CHF manifested by pleural and pericardial effusion. Thoracic radiographs confirmed pleural effusion, and echocardiography identified pericardial effusion with a hypertrophic cardiomyopathy (HCM) phenotype and left atrial enlargement, accompanied by severe azotemia on laboratory testing. Pleural effusion was managed initially with thoracocentesis for respiratory palliation, achieving partial drainage. Because escalation of loop-diuretic therapy was considered undesirable due to concern for worsening renal function, bexagliflozin (5 mg/cat PO q24h) was initiated with pimobendan as an adjunct/bridge strategy, and subcutaneous fluids were temporarily withheld given concern for fluid overload. During follow-up, serial thoracic radiographs and echocardiography documented progressive improvement culminating in complete resolution of pleural and pericardial effusions; subcutaneous fluid therapy was reintroduced thereafter without recurrence, and the cat has remained clinically stable. Renal indices fluctuated early but subsequently showed a tendency toward stabilization rather than a progressive worsening trajectory. Structured safety monitoring—including serial assessment of urinary glucose, blood glucose, and ketones—did not reveal clinically significant adverse events. This case suggests that, in carefully selected cats with CHF and advanced CKD in which conventional diuretic escalation is high risk, SGLT2i may be considered as an adjunct/bridge option for decongestion, with particular emphasis on proactive monitoring for ketone abnormalities and euglycemic ketoacidosis.

## Introduction

Congestive heart failure (CHF) in cats commonly presents as pleural effusion, pulmonary edema, and/or pericardial effusion, often accompanied by dyspnea and hypoxemia (1–4). Rapid and effective decongestion is therefore a central goal of CHF management (3, 4). Loop diuretics remain the cornerstone of decongestive therapy (4). However, chronic kidney disease (CKD) is frequently concurrent in cats with CHF, and in advanced CKD (IRIS stage 3–4), diuretic therapy may precipitate or exacerbate azotemia, dehydration, and electrolyte derangements (4–6). As a result, cats with coexisting CHF and advanced CKD often face a therapeutic dilemma: clinicians must relieve congestion adequately while preserving renal function (5, 6). In some patients, the tolerability of conventional diuretic strategies may be limited and/or satisfactory decongestion may be difficult to achieve (5, 6). In such scenarios, alternative or adjunctive approaches to decongestion may be warranted.

Sodium–glucose cotransporter 2 inhibitors (SGLT2i) were originally developed for type 2 diabetes mellitus (DM) (7). By inhibiting proximal tubular glucose and sodium reabsorption, SGLT2i increase urinary glucose excretion and promote a mild natriuresis accompanied by osmotic diuresis (7, 8). This pharmacologic profile can facilitate gradual fluid offloading and reduction of filling pressures through enhanced urinary water loss (8). In addition, greater distal sodium delivery may restore tubuloglomerular feedback, thereby lowering intraglomerular pressure—an effect proposed to support renal hemodynamic stability in cardiorenal settings (7). In human medicine, these combined hemodynamic and renal mechanisms have supported the expansion of SGLT2i use in heart failure and CKD populations, including patients without diabetes (8–12). This translational rationale is particularly relevant in veterinary patients, in whom cardiac and renal disease commonly coexist (6, 13, 14).

In veterinary medicine, clinical experience with SGLT2i has largely accumulated in the context of feline DM (14–16), and bexagliflozin is an SGLT2i approved by the United States Food and Drug Administration (FDA) for the treatment of DM in cats (15, 16). In veterinary cardiology—particularly in dogs—emerging reports suggest that SGLT2i may favorably influence cardiac structure and functional indices (17, 18). Nevertheless, clinical reports describing SGLT2i use specifically to achieve decongestion in cats with CHF complicated by advanced CKD remain limited.

We describe the clinical course of a cat with CHF and concurrent advanced CKD (IRIS stage 3–4) in which concern for worsening renal function could restrict the application of loop diuretics, and a low-dose bexagliflozin regimen was implemented as an adjunct/bridge strategy for decongestion. Changes in congestion were objectively documented using serial thoracic radiographs and follow-up echocardiography. Given the presence of advanced CKD, renal trajectory was closely monitored, and structured safety surveillance was performed, including assessment of glycosuria, blood glucose, ketones, and serial electrolyte measurements.

## Case description

An 11-year-old, 4.3 kg, neutered male British Shorthair cat was presented to our hospital for decreased appetite, lethargy, and tachypnea (Day 0). The cat had been diagnosed with chronic kidney disease (CKD) approximately 18 months earlier. As the disease progressed to IRIS stage 3–4 approximately 3 months prior to presentation, the cat had been receiving subcutaneous 0.9% sodium chloride (normal saline; 100 mL per administration) at home, administered by the owner three times per week; this had recently been increased to every 2 days. The cat was also receiving amlodipine and telmisartan for concurrent systemic hypertension and proteinuria. The cat had intermittently received mirtazapine for episodes of hyporexia; however, recent administration failed to restore appetite and activity adequately, prompting referral for further evaluation.

On presentation, the body weight was 4.3 kg, which was increased relative to the most recent owner-reported weight (approximately 3.9 kg). Given the recent history of reduced appetite, fluid retention was considered a potential contributor to the weight gain. Arterial blood gas analysis (PaO2) and pulse oximetry (SpO2) were not assessed at presentation. The respiratory rate was approximately 60 breaths/min and the heart rate was 240 beats/min (reference interval, 140–220 beats/min). No cardiac murmur was auscultated, and heart sounds were subjectively muffled. Rectal temperature was 38.4 °C (reference interval 38.0–39.2 °C), and Doppler systolic blood pressure was 130 mmHg (ACVIM normal 90–139 mmHg).

**Figure 1:**
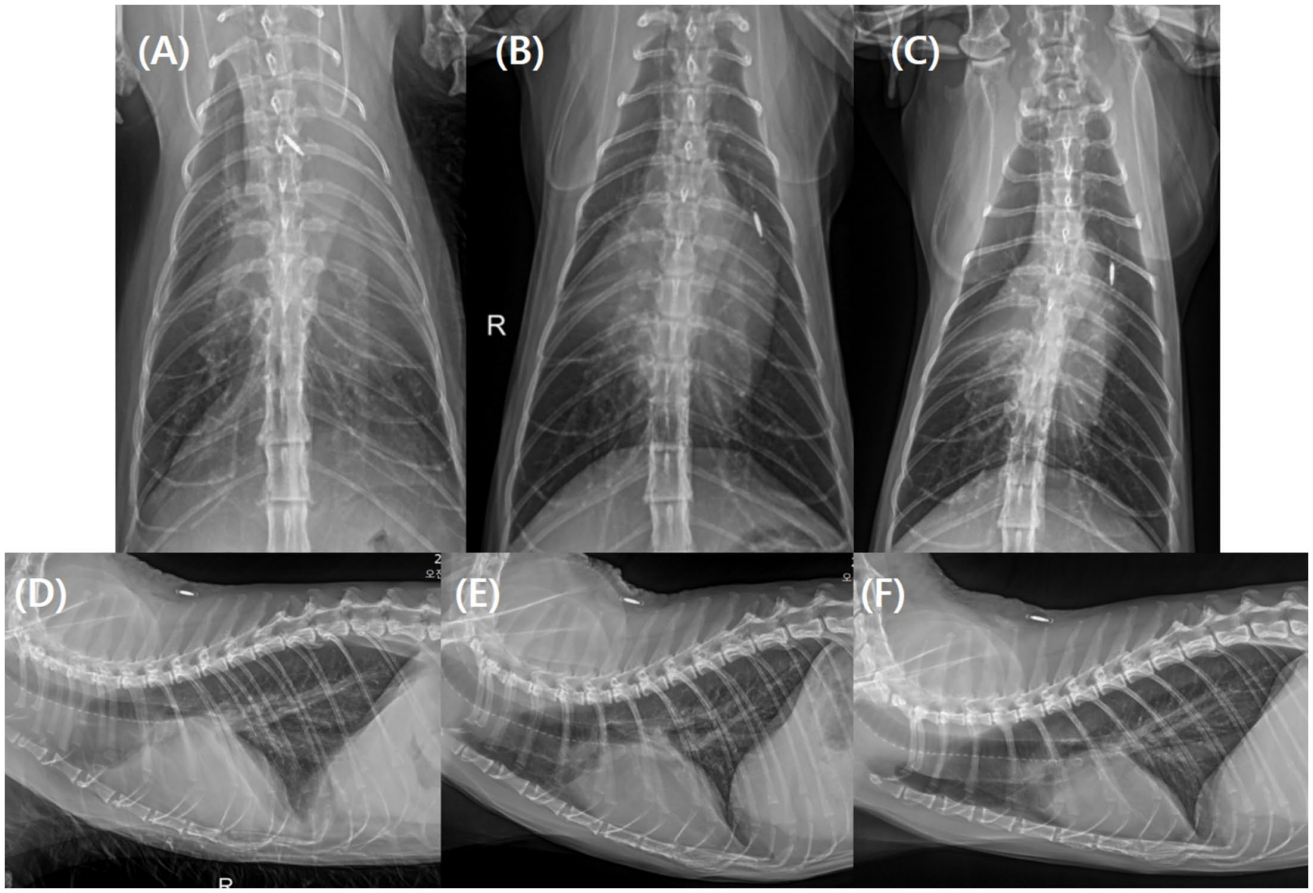
Serial thoracic radiographs documenting resolution of pleural effusion. Ventrodorsal (VD) views are shown in panels **(A–C)**, and lateral views are shown in panels **(D–F)**. Images were obtained immediately after thoracocentesis on Day 0 **(A,D)**, 10 days later **(B,E)**, and 17 days later **(C,F)**. Pleural effusion remained visible on Day 0 after partial thoracocentesis and decreased progressively thereafter, with complete radiographic resolution by Day 17. Subsequent recheck radiographs remained unchanged from Day 17, with no recurrence of pleural effusion during the observation period.

Thoracic radiographs confirmed pleural effusion, which was most prominent in the left cranial to middle lung fields and the right cranial lung field (Figure 1). A targeted (limited) echocardiographic examination was performed on Day 0 to rapidly assess cardiac structure and the presence of effusions in the setting of respiratory distress. Echocardiography identified concurrent pericardial effusion and revealed left ventricular hypertrophy with left atrial enlargement. Specifically, diastolic left ventricular wall thickness was 6.5 mm (reference < 5.5 mm), and LA/Ao was 1.80 (reference < 1.5) on the right parasternal short-axis view. To further characterize left atrial (LA) phasic function at presentation, LA fractional shortening (LA FS) was 14.3%. Marked tachycardia and respiratory compromise at presentation resulted in fusion of the transmitral E and A waves, limiting diastolic Doppler assessment. The peak velocity of the fused transmitral inflow (E/A) was 1.62 m/s. Pulsed-wave tissue Doppler imaging at the medial (septal) mitral annulus yielded e′ 4.97 cm/s, a′ 9.55 cm/s, and s′ 6.47 cm/s. Pulmonary venous flow was not evaluated due to patient instability. Spontaneous echo contrast was not observed. Left ventricular outflow tract obstruction, including systolic anterior motion (SAM), was not identified on 2D and color Doppler assessment. Collectively, these findings were most suggestive of a hypertrophic cardiomyopathy (HCM) phenotype; however, restrictive cardiomyopathy (RCM) could not be completely excluded because comprehensive diastolic indices could not be adequately assessed at presentation. According to the referring medical record, a recent total thyroxine (T4) concentration was 2.5 μg/dL (reference interval, 0.9–3.7 μg/dL), making hyperthyroidism less likely as a cause of secondary left ventricular hypertrophy. Overall, congestive heart failure (CHF) secondary to cardiomyopathy was suspected, and the pleural and pericardial effusions were considered compatible with this congestive state.

A complete blood count (CBC) was performed on Day 0 and was within reference intervals (WBC 5.6 × 10^9/L; reference interval 5.5–19.5; HCT 30.1%; reference interval 26–51; platelets 262 × 10^9/L; reference interval 100–518). Serum biochemistry results included BUN 98 mg/dL (reference interval 17.6–32.8), creatinine 5.78 mg/dL (reference interval 0.8–1.8), phosphorus 10.9 mg/dL (reference interval 2.6–6.0), glucose 121 mg/dL (reference interval 71–148), total protein 7.0 g/dL (reference interval 5.7–7.8), albumin 3.2 g/dL (reference interval 2.3–3.5), globulin 3.8 g/dL (reference interval 2.7–5.2), ALT 49 U/L (reference interval 22–84), ALKP 78 U/L (reference interval 38–165), calcium 9.8 mg/dL (reference interval 8.8–11.9), and feline pancreatic lipase (fPL) 2.6 ng/mL (reference interval 0–3.5). Blood ketones (*β*-hydroxybutyrate) were 0.3 mmol/L, measured using a handheld meter, and urine dipstick testing was negative for both ketones and glucose at presentation. Serum electrolytes were sodium (Na) 150 mmol/L, potassium (K) 4.3 mmol/L, and chloride (Cl) 124 mmol/L (reference intervals: Na 147–162, K 2.9–4.2, Cl 112–129). Venous blood gas analysis showed no clinically relevant abnormalities.

Based on clinical signs and imaging findings, thoracocentesis was performed and approximately 60 mL of pleural fluid was partially removed for respiratory palliation. Following drainage, the respiratory rate decreased to approximately 40 breaths/min. The pleural fluid appeared grossly clear, and fluid analysis (total protein 2.1 g/dL; TNCC 0.35 × 10^9^/L) was consistent with a transudate based on conventional criteria (total protein <2.5 g/dL and low TNCC).

Because the cat had advanced CKD with severe azotemia, aggressive decongestion using a conventional loop-diuretic–based approach was considered likely to increase the risk of worsening renal function. After discussion with the owner, pimobendan (0.25 mg/kg PO q12h) and bexagliflozin (5 mg/cat PO q24h; prepared by dividing a commercially available 15-mg tablet into thirds) were initiated as an adjunct/bridge strategy for decongestion. Pimobendan was selected primarily to support forward flow in the setting of cardiomyopathy-associated CHF with echocardiographic evidence suggestive of reduced left atrial systolic function. Subcutaneous fluid administration was temporarily discontinued due to concern for exacerbation of volume overload. Amlodipine and telmisartan were continued unchanged throughout the observation period, and the diet was maintained without modification. Because reduced caloric intake can increase the risk of ketosis during SGLT2i therapy, mirtazapine was administered as needed during episodes of hyporexia, and the cat was able to maintain voluntary food intake thereafter. Hydration status was closely monitored throughout treatment.

**Figure 2:**
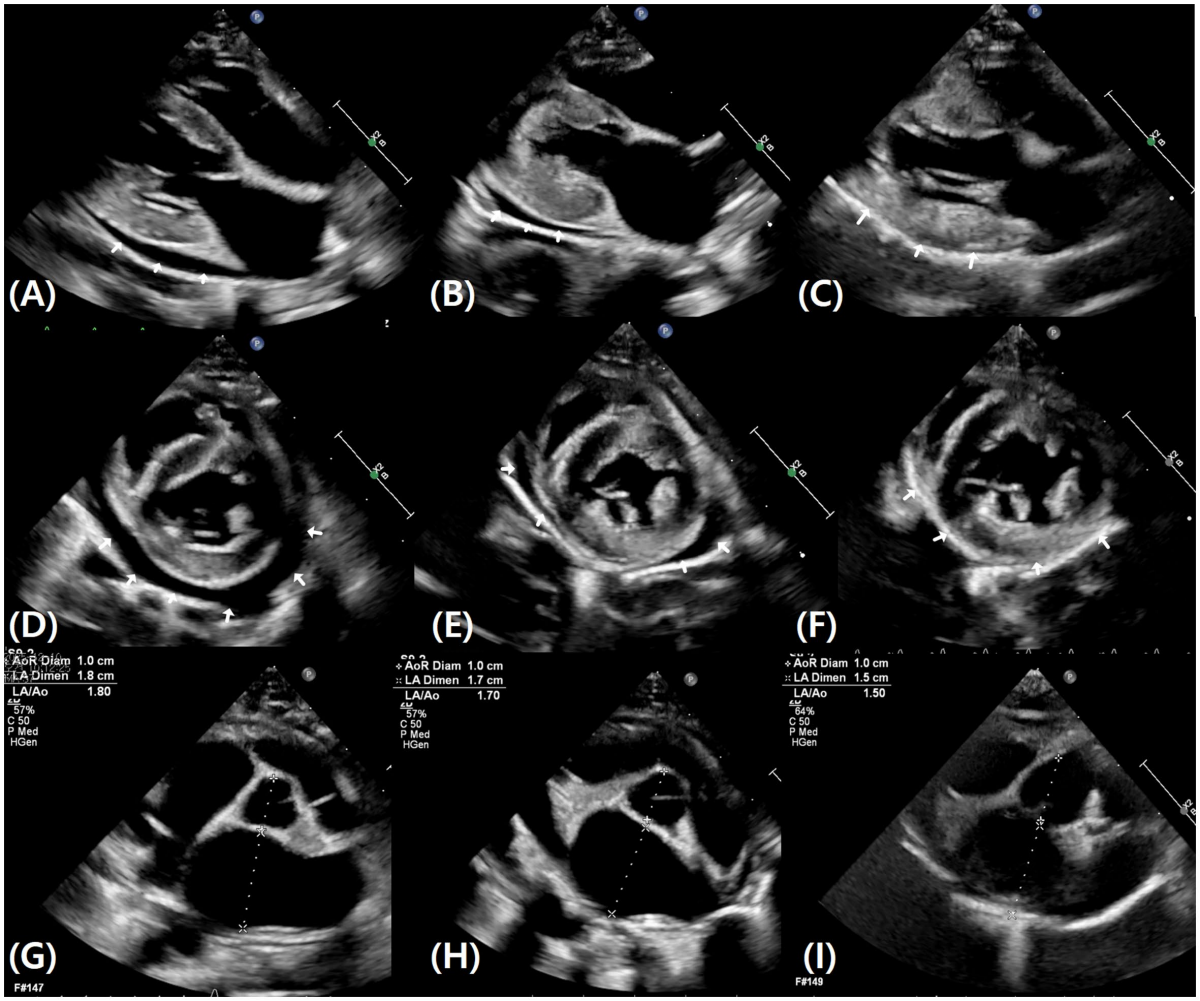
Serial echocardiographic images demonstrating resolution of pericardial effusion and reduction in left atrial size. Right parasternal long-axis views are shown in panels **(A–C)**, and right parasternal short-axis views are shown in panels **(D–F)**. White arrows indicate the presence or absence of pericardial effusion. Images were obtained on Day 0 **(A,D)**, Day 10 **(B,E)**, and Day 17 **(C,F)**. Pericardial effusion decreased progressively from Day 0 to Day 10 and was no longer visible by Day 17. Panels **(G–I)** show right parasternal short-axis views used for left atrial-to-aortic ratio (LA/Ao) measurement on Day 0 **(G)**, Day 10 **(H)**, and Day 17 **(I)**, demonstrating a reduction in LA/Ao over time (Day 0, 1.80; Day 10, 1.70; Day 17, 1.50).

Over the subsequent 39 days, the cat was re-evaluated on five occasions, with monitoring of body weight, imaging (thoracic radiography and echocardiography), serial blood tests (renal indices and electrolytes), urinalysis, and ketones (blood and urine) (Table 1). Although urine output and water intake were not quantitatively recorded, the owner subjectively reported a mild increase in water intake and litter box use after initiation of bexagliflozin. Body weight gradually decreased after presentation and stabilized between 3.7 and 3.8 kg from Day 10 onward. Serial thoracic radiographs demonstrated progressive reduction in pleural effusion, with no radiographically apparent pleural effusion from Day 17 (Figure 1). Follow-up echocardiography similarly confirmed complete resolution of pericardial effusion from Day 17 (Figure 2). Serial echocardiography also demonstrated a reduction in left atrial size, with LA/Ao decreasing from 1.80 (Day 0) to 1.70 (Day 10) and 1.50 (Day 17) (Figure 2). After decongestion was confirmed (Day 17), subcutaneous fluids were reintroduced to support CKD management and hydration (lactated Ringer’s solution, 100 mL SC twice weekly). Following re-initiation, body weight remained stable within the same range, and respiratory status remained clinically stable.

**Table 1:** Longitudinal monitoring of renal indices, electrolytes, safety variables, clinical parameters, imaging findings, and fluid-management procedures in a cat with congestive heart failure (CHF) and advanced chronic kidney disease (CKD) treated with bexagliflozin as an adjunct/bridge decongestive strategy.

Variable	Day 0	Day 10	Day 17	Day 31	Day 39
Renal/laboratory					
BUN (mg/dL) (RI 17.6–32.8)	98.0	104.3	107.1	102.1	103.9
Creatinine (mg/dL) (RI 0.8–1.8)	5.78	6.34	6.17	6.05	5.97
Phosphorus (mg/dL) (RI 2.6–6)	10.9	11.5	11.7	11.4	11.1
Electrolyte/laboratory					
Sodium (mmol/L) (RI 147–162)	150	152	152	151	152
Potassium (mmol/L) (RI 2.9–4.2)	4.3	4.5	4.5	4.4	4.5
Chlorine (mmol/L) (RI 112–129)	124	118	119	125	119
Safety monitoring					
Blood glucose (mg/dL) (RI 71–148)	121	120	115	119	118
Blood ketones (mmol/L)	0.3	0.4	0.4	0.3	0.4
Urine glucose (dipstick)	Negative	2+	2+	2+	2+
Urine ketones (dipstick)	Negative	Negative	Negative	Negative	Negative
Urine WBC (sediment microscopy)	0/HPF	0/HPF	0/HPF	0/HPF	0/HPF
Bacteriuria (sediment microscopy)	None seen	None seen	None seen	None seen	None seen
Clinical parameters					
Body weight (kg)	4.3	4.0	3.8	3.7	3.8
Imaging					
Pleural effusion (thoracic radiographs)	Present	Decreasing	Resolved	No recurrence	No recurrence
Pericardial effusion (echocardiography)	Present	Decreasing	Resolved	No recurrence	No recurrence
Procedures/therapy					
Thoracocentesis (mL)	60				
Subcutaneous fluids	Withheld	Withheld	Restarted	Yes	Yes

Renal trajectory was assessed using serial blood tests (BUN, creatinine, and phosphorus). Electrolyte status was monitored using serial measurements of sodium, potassium, and chloride. Safety monitoring for SGLT2 inhibition included blood glucose, blood ketones, urine glucose (dipstick), and urine ketones (dipstick), along with urine sediment evaluation for evidence of cystitis/urinary tract infection (urine WBC and bacteriuria). Congestion dynamics were tracked by thoracic radiography (pleural effusion) and echocardiography (pericardial effusion). The table also summarizes thoracocentesis performed for respiratory palliation and the timing of subcutaneous fluid discontinuation and reintroduction. RI, reference interval; HPF, high-power field; BUN, blood urea nitrogen; WBC, white blood cells.

Renal indices (BUN and creatinine) showed mild early increases after presentation and then fluctuated within a relatively stable range without a progressive worsening trajectory (Table 1). Safety monitoring demonstrated that blood ketones remained between 0.3 and 0.4 mmol/L throughout follow-up, while urine ketones remained negative on dipstick testing. Serial blood glucose measurements remained within the euglycemic range throughout follow-up. Urine dipstick testing consistently detected glucosuria (2+), confirming the expected pharmacodynamic effect of SGLT2i. Serum sodium and chloride remained within the reference intervals throughout follow-up, while potassium was persistently mildly increased (4.3–4.5 mmol/L) without clinically apparent sequelae. At the time of reporting (Day 39), appetite and activity were improved compared with presentation, and the cat remained clinically stable without recurrence of radiographic or echocardiographic evidence of congestion.

## Discussion

To the authors’ knowledge, this is the first report describing the use of low-dose bexagliflozin as an adjunct/bridge decongestion strategy in a cat with CHF complicated by advanced CKD. In this case, loop-diuretic therapy was considered limited by concern for worsening renal function, and renal trajectory and structured safety monitoring were concurrently implemented.

This case highlights a clinically important and therapeutically challenging scenario: development of CHF with pleural and pericardial effusion in a cat with advanced CKD (IRIS stage 3–4) (1–3). In such patients, standard loop-diuretic–based decongestion may be constrained by the risk of exacerbating azotemia, dehydration, and electrolyte abnormalities, creating a therapeutic dilemma between achieving adequate decongestion and preserving renal function (4–6). In the present case, after initiating low-dose bexagliflozin (5 mg/cat PO q24h) in combination with pimobendan as an adjunct/bridge approach, a clear improvement in fluid overload was observed during follow-up. Serial thoracic radiographs demonstrated gradual reduction and eventual resolution of pleural effusion (Figure 1), and follow-up echocardiography confirmed complete resolution of pericardial effusion (Figure 2), supporting clinically meaningful decongestion. In addition, body weight at presentation exceeded the owner-reported recent weight despite reduced appetite, raising suspicion for fluid retention; subsequent weight loss followed by stable maintenance paralleled the imaging-confirmed resolution of congestion and clinical stabilization. Because body weight can be influenced by caloric intake and hydration status, it was not interpreted as a standalone indicator, but rather considered alongside imaging findings and respiratory status.

Given its glucose-driven osmotic diuretic/natriuretic profile, SGLT2i has been proposed to support decongestion through mechanisms that may differ from conventional loop diuretics (8, 19–23). In this patient, several monitored findings were consistent with target engagement and supported a plausible contribution of SGLT2i to decongestion. Serial blood glucose measurements remained euglycemic and blood *β*-hydroxybutyrate remained low (0.3–0.4 mmol/L) with negative urine ketones, supporting short-term metabolic safety during follow-up. Renal indices and electrolytes were also assessed serially and, after mild early fluctuations, did not show a progressive worsening trajectory throughout follow-up (Table 1). Serial Doppler blood pressure measurements were not consistently obtainable because the cat was highly stress-sensitive during re-evaluations; therefore, blood pressure trends could not be used as a reliable surrogate marker of preload change in this case.

In CHF, where interstitial fluid accumulation (e.g., pulmonary/pleural fluid) often drives clinical signs, this pharmacologic profile provides a physiologic rationale for considering SGLT2i as an adjunct strategy in high-risk renal patients in whom conventional diuretic escalation is undesirable (6, 11–14). In this case, persistent glucosuria on serial urine dipstick testing was consistent with the expected pharmacodynamic effect of SGLT2i (15, 16). Following initiation of therapy, serial imaging documented progressive improvement in congestion, including gradual reduction and radiographic resolution of pleural effusion and complete resolution of pericardial effusion (Figures 1, 2), accompanied by a reduction in left atrial size. Collectively, these findings suggest a temporally associated course in which bexagliflozin administration may have been related to the decongestion process; however, causality cannot be established from a single case.

In this case, pimobendan was used as an adjunct in cardiomyopathy-associated CHF because echocardiography suggested reduced atrial ‘booster pump’ contribution, as reflected by a low left atrial fractional shortening (LA FS = 14.3%). A recent practical evidence-based review on pimobendan use in cats summarizes that its potential clinical effects may extend beyond left ventricular inotropic support and may include favorable effects on left atrial/auricular systolic function and left atrial size in selected cats with CHF (24). On this basis, pimobendan was considered a reasonable adjunct to support atrial contribution to overall cardiac performance and forward flow during the acute congestive episode; however, concurrent pimobendan should be considered when interpreting clinical response in a single case.

The clinical relevance of this case extends beyond improvement in congestion after bexagliflozin administration. Notably, imaging-confirmed resolution of pleural and pericardial effusions occurred in the absence of loop diuretics, and renal indices during follow-up did not show a progressive worsening trajectory; instead, after an early increase, BUN/creatinine values fluctuated and then tended to stabilize (Table 1). Loop diuretics are central to CHF decongestion, but in cats with advanced CKD the risks of dehydration, electrolyte derangements, and worsening azotemia may constrain treatment intensity (4–6). In this case, however, interpretation of renal indices must remain cautious. The early rise and subsequent stabilization cannot be attributed to a single mechanism, as multiple concurrent factors likely contributed, including changes in volume status at presentation, fluid redistribution after thoracocentesis, variation in intake and hydration, and discontinuation of subcutaneous fluids for 17 days followed by reintroduction. Similarly, electrolyte values should be interpreted in context; sodium and chloride remained within the reference intervals, whereas potassium was persistently mildly increased, likely reflecting the patient’s advanced CKD and without evidence of clinically relevant electrolyte complications during follow-up. In addition, renal indices in CHF can fluctuate with hemodynamic shifts and volume status, and the cardiorenal concept—including the potential influence of congestion itself (including venous congestion) on renal function—should be considered (5, 6). Accordingly, the renal trends observed here are best interpreted within the overall clinical course and the accompanying fluid-management strategy, rather than as evidence of a specific mechanistic renal benefit. Nevertheless, the absence of a sustained worsening trend and the later stabilization pattern support the hypothesis that SGLT2i could be explored as an adjunct option when selecting decongestion strategies in cats with CHF and advanced CKD (13, 14).

Importantly, the potential benefits of SGLT2i may not be limited to osmotic diuresis. In human medicine, pleiotropic mechanisms have been proposed, including improved myocardial energetic efficiency, modulation of sympathetic activity, anti-inflammatory and anti-fibrotic effects, favorable cardiac remodeling, and nephroprotective pathways (8, 19–22, 25). However, the extent to which any of these mechanisms contributed to the clinical course of this single feline case cannot be determined, and reproducibility and effect size in non-diabetic cats with CHF and advanced CKD remain unclear. Prospective studies will be required to validate both mechanistic and clinical utility in comparable veterinary populations (13, 14).

In veterinary medicine, most clinical experience with SGLT2i has been accumulated through their approved use for feline DM (14–16), and studies in veterinary cardiology—particularly in dogs—have suggested that SGLT2i may favorably influence cardiac structure and functional indices (17, 18). Despite this, clinical reports describing SGLT2i specifically as a decongestion strategy in cats with concurrent CHF and advanced CKD remain extremely limited, leaving uncertainty regarding patient selection, dose optimization, and monitoring strategies (13, 14). By providing imaging-based documentation of congestion dynamics and an approach incorporating renal trajectory and structured safety monitoring, this report partially addresses this knowledge gap.

In the present case, the therapeutic goal was decongestion rather than glycemic control. Bexagliflozin induces glucosuria via SGLT2i, and available feline data suggest that the magnitude of glucosuria may increase with higher doses, with the labeled regimen (15 mg/cat) having been developed to improve glycemic control in diabetic cats (15). However, direct extrapolation of the labeled diabetic dose to a high-risk, non-diabetic cat with cardiomyopathy-associated CHF and advanced CKD is inherently limited (13, 14). Therefore, 5 mg/cat PO q24h was selected as a conservative starting dose rather than an evidence-based target dose for decongestion. This choice was intended to balance (i) achieving measurable glucosuria as a practical indicator of target engagement in an off-label setting and (ii) minimizing the risk of excessive osmotic diuresis and dehydration/worsening azotemia in advanced CKD, as well as ketone-related adverse events (including euDKA) (13, 14, 26). In addition, a technical disclosure describing a non-linear glucosuria dose–response (with a steep increase at lower doses and an apparent plateau at higher doses) further supported initiating therapy at a reduced starting dose (27). This dose selection does not imply that lower doses are inherently safer or more effective; the optimal dose, dose–response relationship, and combination strategies for non-diabetic cardiac indications remain to be defined (13, 14).

Clinically important safety concerns have been reported in cats receiving SGLT2i, particularly euglycemic diabetic ketoacidosis (euDKA) (13, 14, 16). Because euDKA may occur without marked hyperglycemia, it can be clinically overlooked and may begin with nonspecific signs such as hyporexia, vomiting, lethargy, and dehydration (13, 14, 27). Accordingly, careful patient selection and structured monitoring are essential when using SGLT2i in cats (13, 14, 16). Monitoring should include assessment for gastrointestinal intolerance, evidence of urinary tract infection (including urine sediment evaluation), hypoglycemia, and the risk of euDKA, with paired monitoring of blood glucose and blood and/or urine ketones, in addition to renal indices and electrolytes (13, 14, 16, 28). In this case, structured monitoring over 39 days included blood glucose, urine glucose, blood/urine ketones, renal indices, electrolytes, and body weight, and no clinically significant adverse effects were observed during the observation period.

This report has several limitations. First, the observed improvement in congestion cannot be attributed to bexagliflozin alone. Thoracocentesis, concurrent pimobendan administration, temporary discontinuation and later reintroduction of subcutaneous fluids, the natural course of the underlying disease, and changes in intake and hydration could all have influenced both congestion and renal indices. In addition, the temporal overlap between thoracocentesis and initiation of therapy introduces potential attribution bias, as rapid short-term improvement after drainage could be over-ascribed to medication effects. Second, as a single case report, generalizability is limited, and the follow-up period was relatively short, precluding evaluation of long-term outcomes such as recurrent congestion, survival, and renal endpoints. Third, SDMA was not measured due to owner-related financial constraints; therefore, renal status was assessed primarily using serial creatinine/BUN and urinalysis, and subtle changes in glomerular filtration may have been missed. Fourth, quantitative assessment of congestion and pharmacologic effect was limited; standardized respiratory metrics, urine output, and quantitative urinary glucose excretion were not prospectively standardized, restricting quantitative comparison of the magnitude of decongestion. Fifth, although no major adverse events were observed, rare but clinically significant class-associated complications (e.g., euDKA) cannot be considered unlikely based on short-term single-case observation (13, 14, 16, 26). Finally, the optimal dose, dose–response relationship, combination therapy strategy, and the influence of CKD on drug exposure and response in non-diabetic cats with CHF remain undefined (13, 14, 26). Prospective controlled studies in cats with non-diabetic CHF and concurrent CKD are needed to systematically evaluate efficacy and safety, determine optimal dosing and combination strategies, and establish practical monitoring protocols.

## Conclusion

This case suggests that low-dose bexagliflozin, used as an adjunct/bridge strategy, may support decongestion in a cat with CHF and advanced CKD when conventional loop-diuretic escalation is considered high risk. Serial radiography and echocardiography documented complete resolution of pleural and pericardial effusions without loop diuretics, while renal indices showed early fluctuation followed by relative stabilization. Structured safety monitoring—including serial blood glucose and ketone assessment—is essential, particularly to mitigate the risk of euglycemic ketoacidosis. Further controlled studies are needed to define efficacy, optimal dosing, and standardized monitoring protocols in feline cardiorenal disease.

## Data Availability

The raw data supporting the conclusions of this article will be made available by the authors, without undue reservation.
