# Supplementary figures and images for: Case Report: Bexagliflozin as an adjunct decongestive strategy in a cat with congestive heart failure and advanced chronic kidney disease
Source: Front Vet Sci. 2026 Mar 13;13:1791139. doi: 10.3389/fvets.2026.1791139 (PMC13021453; doi:10.3389/fvets.2026.1791139)

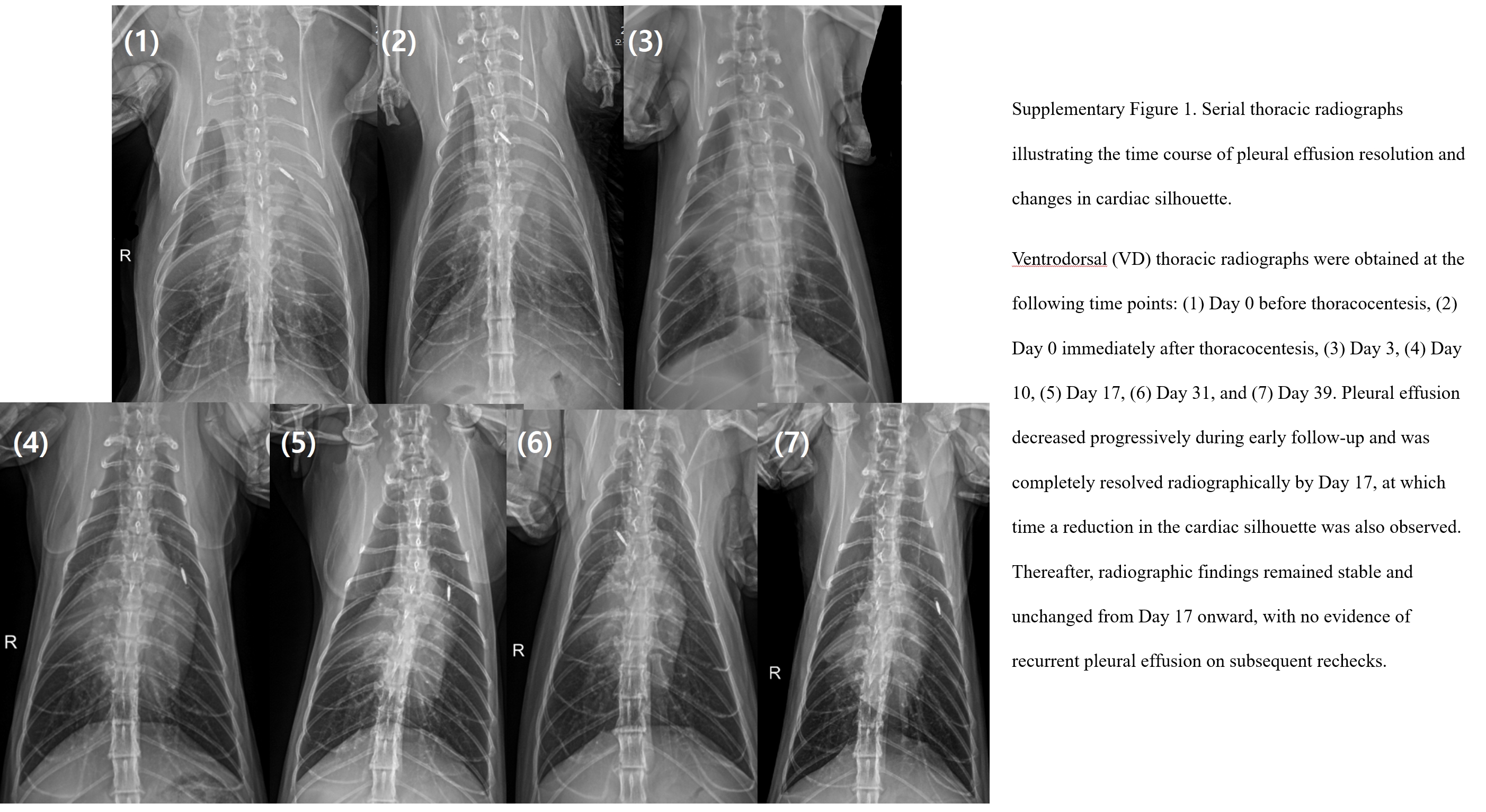

Supplement: Supplementary file 1 [file Image_1.jpeg]

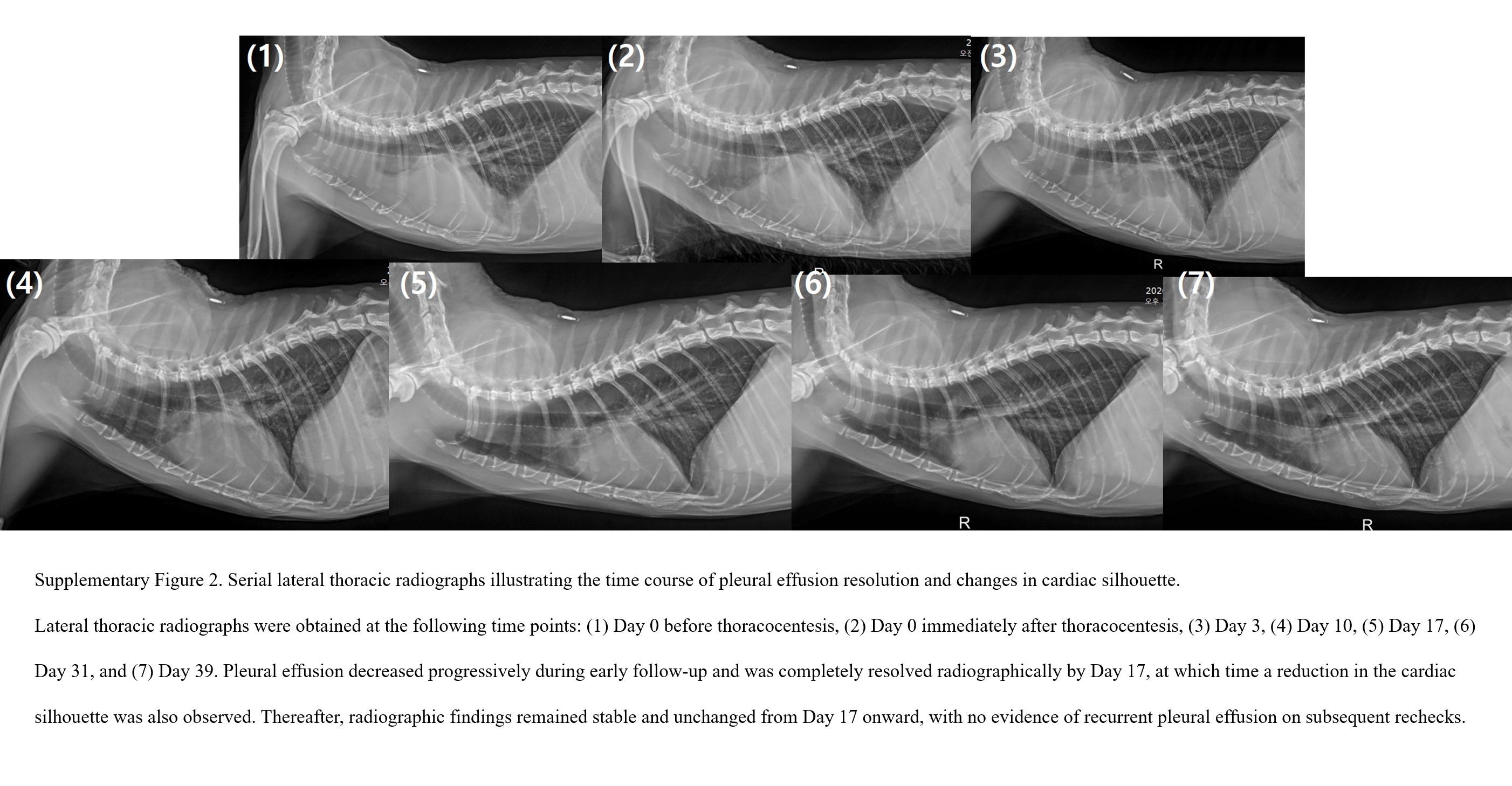

Supplement: Supplementary file 2 [file Image_2.jpeg]

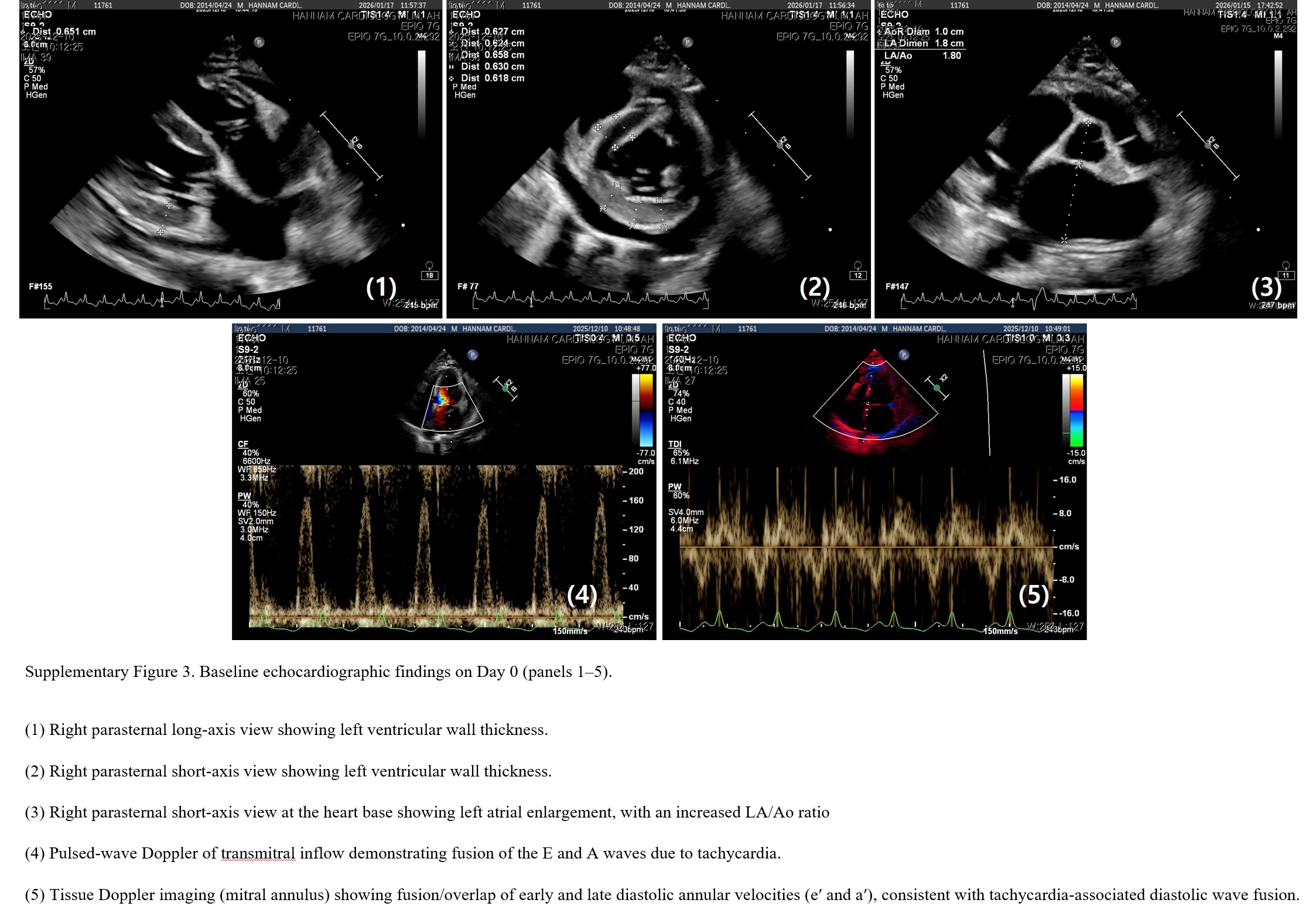

Supplement: Supplementary file 3 [file Image_3.jpeg]
